# Oral Human Papillomavirus Infection in Children during the First 6 Years of Life, Finland

**DOI:** 10.3201/eid2703.202721

**Published:** 2021-03

**Authors:** Stina Syrjänen, Marjut Rintala, Marja Sarkola, Jaana Willberg, Jaana Rautava, Hanna Koskimaa, Anna Paaso, Kari Syrjänen, Seija Grénman, Karolina Louvanto

**Affiliations:** University of Turku, Turku, Finland (S. Syrjänen, M. Rintala, J. Willberg, J. Rautava, H. Koskimaa, A. Paaso, S. Grenman, K. Louvanto);; Central Hospital of Lahti, Lahti, Finland (M. Sarkola); Biohit Oyj, Helsinki, Finland (K. Syrjänen)

**Keywords:** human papillomavirus, HPV, viruses, oral infection, sexually transmitted infections, children, oral cavity, Finland

## Abstract

Human papillomavirus (HPV) infections are found in children, but transmission modes and outcomes are incompletely understood. We evaluated oral samples from 331 children in Finland who participated in the Finnish Family HPV Study from birth during 9 follow-up visits (mean time 51.9 months). We tested samples for 24 HPV genotypes. Oral HPV prevalence for children varied from 8.7% (at a 36-month visit) to 22.8% (at birth), and 18 HPV genotypes were identified. HPV16 was the most prevalent type to persist, followed by HPV18, HPV33, and HPV6. Persistent, oral, high-risk HPV infection for children was associated with oral HPV carriage of the mother at birth and seroconversion of the mother to high-risk HPV during follow-up (odds ratio 1.60–1.92, 95% CI 1.02–2.74). Children acquire their first oral HPV infection at an early age. The HPV status of the mother has a major impact on the outcome of oral HPV persistence for her offspring.

Cutaneous warts are common in children and are acquired mostly through horizontal transmission, but also through vertical transmission; lesions can persist asymptomatically for years (*1*). Unlike human papillomavirus (HPV) infections of the skin, mucosal HPV infections have mostly been regarded as sexually transmitted diseases. However, certain mucosal HPVs (α-HPVs) have also been found in virgins, infants, and children in oral and genital mucosa, implicating a nonsexual mode of transmission (*2*–*6*). From the clinical point of view, virus clades 7, 9, and 10, which include high-risk HPV genotypes, are the major subgroups of α-HPVs. These high-risk HPVs are known to be involved in development of anogenital and head and neck cancers, and estimated to be causally associated with ≈4.5% of all human cancers (*7*).

Nonsexual HPV transmission modes includes vertical or horizontal transmission and autoinoculation (i.e., multisite HPV infections, which can spread from 1 site to another within an individual). Vertical transmission can be further categorized as periconceptual (time around fertilization), prenatal (during pregnancy), and perinatal (during birth or immediately thereafter) (*3*,*8*). Perinatal transmission has been regarded as the most likely explanation for HPV detection in newborns. Several studies have shown that children born to HPV-positive mothers have a higher risk of becoming HPV positive (*9*–*14*). Meta-analysis of 3,128 mother–child pairs showed that children born to HPV-positive mothers were 33% more likely to be HPV positive than children born to HPV-negative mothers (*6*). This risk was even higher (45%) when only high-risk HPV infections were considered (*6*).

Periconceptual transmission could theoretically occur through infected oocytes or spermatozoa. HPV DNA has been detected in semen, sperm, seminal plasma, spermatozoa, and vas deferens (*15*). Studies have shown that the placenta is not a sterile microenvironment; instead, it has been shown to harbor both viruses and bacteria, which can further influence the maternal part of periconceptual transmission (*16*). HPV has been found in the placenta and shown to replicate in trophoblasts, which could feasibly explain prenatal transmission (*2*). A recent systematic review on intrauterine HPV transmission showed that the pooled percentage of antenatal vertical HPV transmission was 4.9% (95% CI 1.65%–9.85%), and the mode of delivery had no effect on this transmission (*17*).

Elucidation of the early HPV infections is needed to generate a comprehensive overview on the natural history of HPV infections. The main aims of this study were to characterize oral HPV prevalence and genotype variation in children in Finland and determine infection outcomes during the first 6 years of life.

## Material and Methods

### Participants

The Finnish Family HPV Study is a prospective cohort study conducted at the University of Turku and Turku University Hospital, Turku, Finland, since 1998. Members of 329 families were enrolled (329 mothers, 131 fathers, and 331 newborns) as described (*13*,*14*,*18*). Women were enrolled at a minimum of 36 weeks of their index pregnancy and subsequently followed up for 6 years. HPV status of mothers was not available before enrollment. All parents provided written, informed consent at the first visit for the study. The Research Ethics Committee of Turku University Hospital approved the study protocol and its amendments (#2/1998 and #2/2006).

We collected demographic data from parents by using structured questionnaires at baseline and at 3-year and 6-year visits. General health of children was recorded at the 36-month visit (Appendix Table 1), and examination of oral mucosa was performed at the 6-year follow-up visit.

**Table 1:** Prevalence and genotype variation of oral HPV infections among 324 children from birth to 6 y of age*

Variable	At birth	3 d	1 mo	2 mo	6 mo	12 mo	24 mo	36 mo	6 y
Oral sample	324	309	300	296	295	291	264	265	201
Mean ± SD age, mo	0	0.08 ± 0.37	1.14 ± 0.18	2.23 ± 0.33	6.41 ± 0.45	12.61 ± 0.67	24.90 ± 1.01	36.98 ± 1.31	77.47 ± 11.01
Any HPV+	74 (22.8)	41 (13.3)	57 (19)	48 (16.2)	44 (14.9)	34 (11.7)	25 (9.5)	23 (8.7)	41 (20.4)
Girls, any HPV+	35 (47.9)	19 (46.3)	25 (43.9)	29 (60.4)	20 (46.5)	20 (58.5)	14 (56.0)	10 (43.5)	20 (49.9)
Boys, any HPV+	39 (52.1)	22 (53.7)	32 (56.1)	19 (39.6)	24 (53.5)	14 (51.5)	9 (44.0)	13 (56.5)	21 (51.2)
Single HPV+	62 (19.1)	36 (11.7)	55 (18.3)	44 (14.8)	36 (12.2)	33 (11.0)	20(7.6)	21 (7.9)	35 (17.4)
Multiple HPV (>2)	12 (3.7)	5 (1.6)	2 (0.7)	4 (1.4)	8 (2.7)	1 (0.3)	4 (1.5)	2 (0.8)	6 (3.0)
No. HPV genotypes	15	5	8	7	9	10	7	4	8
Low-risk HPV									
HPV6	11 (3.4)	5 (1.6)	3 (1.0)	7 (2.4)	4 (1.4)	3 (1.0)	1 (0.4)	2 (0.8)	4(2.0)
HPV11	1(0.3)	–	1 (1.0)	5 (1.7)	4 (1.4)	1 (0.3)	–	1 (0.4)	–
HPV70	2 (0.6)	–	–	1 (0.3)	1 (0.3)	1 (0.3)	2 (0.8)	–	–
High-risk HPV									
HPV16	25 (7.7)	28 (9.1)	30 (10)	16 (5.4)	14 (4.7)	14 (4.8)	9 (3.4)	17 (6.4)	20(10)
HPV18	4 (1.2)	1 (0.3)	9 (3.0)	11 (3.7)	7 (2.4)	8 (2.7)	4 (1.5)	1 (0.4)	2 (1.0)
HPV31	31 (0.9)	–	–	2 (0.7)	–	1 (0.3)	–	–	3 (1.5)
HPV33	2 (0.6)	–	8 (2.7)	2 (0.7)	1 (0.3)	–	2 (0.8)	–	2 (1.0)
HPV39	1 (0.3)	–	1 (0.3)	–	–	–	–	–	2 (1.0)
HPV45	1 (0.3)	–	1 (0.3)	–	1 (0.3)	–	–	–	1 (0.5)
HPV51	–	–	–	–	1 (0.3)	1 (0.3)	–	–	–
HPV52	–	–	–	–	–	1 (0.3)	–	–	–
HPV53	1 (0.3)	1 (0.3)	–		–	…	–	–	–
HPV56	3 (0.9)	–	–	–	–	1 (0.3)	1 (0.4)	–	–
HPV58	2 (0.6)	–	–	–	–	1 (0.3)	–	–	1 (0.5)
HPV66	4 (1.2)	1 (0.3)	2 (0.7)	–	1 (0.3)	–	1 (0.4)	–	–
HPV68	1 (0.3)	–	–	–	–	–	–	–	–
HPV82	1 (0.3)	–	–	–	–	–	–	–	–

*Values are no. (%) unless indicated otherwise. Two cases where excluded because of missing sample at birth. HPV, human papillomavirus. –, HPV genotype not found at that visit.

### Samples and HPV Genotyping

Oral scrapings for HPV testing were obtained at birth; at day 3 before leaving the hospital; and at 1-, 2-, 6-, 12-, 24-, and 36-month and 6-year follow-up visits. Oral scrapings were obtained by using a brush (Cytobrush; MedScan Medical AB, https://www.diapath.com) and covering the entire oral mucosa as described (*13*). HPV DNA was extracted from oral scrapings by using the high salt method, as described (*13*). For HPV testing, we used nested PCR (MY09/MY11 external primers and GP05+/bioGP06+ internal primers) because the viral load/cell and the number of infected cells among uninfected cells was expected to be low in oral samples.

After nested PCR, HPV genotyping was performed by using the Multimetrix Kit (Progen Biotechnik GmbH, https://www.progen.com), which detected 24 low-risk, putative high-risk, and high-risk HPV genotypes as follows: 6 low-risk genotypes (HPV6, 11, 42, 43, 44, and 70); 3 putative high-risk genotypes (HPV26, 53 and 66); and 15 high-risk genotypes (HPV16, 18, 31, 33, 35, 39, 45, 51, 52, 56, 58, 59, 68, 73, and 82 (*19*). Blood samples from the mother and father were taken at baseline and at 12, 24, and 36 months of the follow-up and stored as described (*20*). Antibodies to the major capsid protein L1 of HPV6, 11, 16, 18, and 45 were analyzed by using multiplex HPV serologic analysis based on glutathione S-transferase fusion protein capture on fluorescent beads, as described (*21*). Serum samples were scored as positive when antigen-specific medium fluorescence intensity values exceeded the cutoff level of 200 for L1 antigen of individual HPV types.

### Statistical Analysis

Times in months to incident oral HPV infections were calculated from the baseline visit to the first incident event. Genotype-specific HPV persistence was recorded whenever >2 consecutive follow-up samples were positive for the same individual HPV genotype as a single infection or as part of a multiple-type infection. Clearance was defined as an event at any follow-up visit for which a previously HPV-positive test result turned out to be negative and remained HPV negative to the end of the follow-up. Times in months to the first clearance event were calculated as the time of the first visit by an HPV-positive patient to the first clearance event.

Predictors of incident HPV infection and genotype-specific HPV clearance or persistence were analyzed by using the most prevalent high-risk HPV types (species α7: HPV18, 39, 45, 59, 68, and 70; species α9: HPV16, 31 ,33, 35, 52, and 58). To model incident infections and genotype-specific HPV clearance, Poisson regression analysis was used. For persistence, a generalized estimating equation (GEE) modeling was used. In the univariate GEE model, all covariates recorded at baseline and previously implicated as potential risk factors for HPV infections were tested (*13*,*14*). The following risk factors were analyzed for the both parents: age; age at time of first sexual encounter; number of lifetime sexual partners; smoking; use of alcohol; history of skin warts, oral/genital warts, and papillomas; history of sexually transmitted infections; drug consumption; oral and genital HPV DNA status; and HPV serologic results at baseline before the birth of the index child. For the mother, the risk factors were a Pap test at baseline, delivery mode, rupture of membrane, and breast-feeding. In the multivariate GEE model, only variables that were significant in the univariate model were entered and adjusted for age. All statistical tests performed were 2-sided, and a p value <0.05 indicated significance. Statistical analyses were performed by using SPSS (https://www.ibm.com) and Stata version 15 (https://www.stata.com) software packages.

## Results

Our study focused on oral HPV infections among the 331 infants born to the 329 mothers in the Finnish Family HPV Study cohort. The mean ± SD age of the mothers at enrollment was 25.5 ± 3.35 years. Of the 331 newborns, 5 did not participate in oral samplings at any visit, and 2 others had only 1 visit, resulting in a longitudinal cohort of 324 (171 girls and 153 boys) children (Figure). Participants had a follow-up mean ± SD age of 51.9 ± 28.9 months (range 0.03–99.7 months). Of these children, 77.6% were born by vaginal delivery and 22.4% by cesarean section. 

**Figure:**
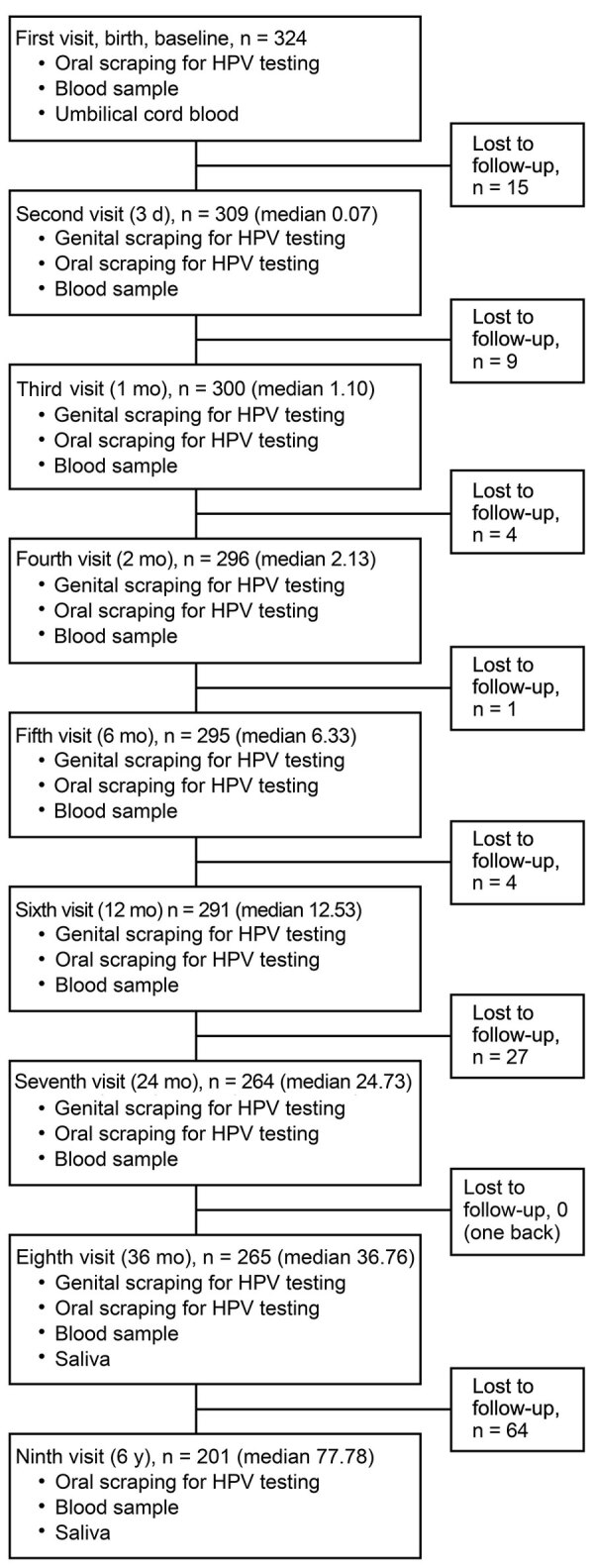
Oral HPV infection in 324 children in the Finnish Family HPV Study during the first 6 years of life. Each visit shows the number of children who participated in the specific follow-up, timeline of the visit, and samples obtained at each visit. HPV, human papillomavirus.

We collected general background information for the general health of the children recorded at the 36-month visit as given by their parents (Table 1). Hand warts were reported only for 3 children, and a common childhood viral disease (molluscum contagiosum) was reported in for 18 children (n = 203). Allergy/atopic symptoms were identified in 26.6% of the children at the 36-month visit. For 54% of the index families, the child was a firstborn. At the 6-year follow-up visit, 11% (20/180) of the children had clinical lesions on their oral mucosa. The most common lesions were small hyperplastic lesions (3.9%), aphtous ulcers (2.8%), and red lesions (2.2%). Only 1 child had a papillary lesion; this child was positive for HPV16 at day 3, month 1, and month 24 and subsequently HPV negative at other visits. Hand warts at the time of examination were detected in 3% of the children. There was no correlation recorded between the presence of hand warts and oral HPV at the 6-year visit.

We also provide an overview of oral HPV infections in children who had HPV genotypes and their point prevalence during the follow-up period (Table 1). The prevalence of oral HPV varied from 8.7% to 22.8% over time, and was lowest at the 3-year visit and highest at birth. Altogether, 18 different HPV genotypes were identified in the oral mucosa. HPV16 was the most prevalent genotype, followed by HPV18, 6, 33, and 31. The prevalence of multiple-type infections varied from 0.3% to 3.7%. Overall, 22.9% of the oral samples collected immediately after birth were positive for HPV DNA. At that time point, the genotype distribution was also the widest (15 different HPV types), and the frequency of multiple-type infections was the highest (3.7%). At the 36-month visit, only 8.7% of the oral samples were positive for HPV, and only 4 genotypes (HPV6, 11, 16, and 18) were identified. At the 6-year visit, HPV prevalence increased again to 20.4%, and 8 different HPV genotypes were identified. A total of 25 different combinations of HPV co-infections (with >2 genotypes) were recorded, HPV16 was present in 56% (14/25) of these samples. When analyzed by sex, we found differences in HPV prevalence at 1-, 2-, 12-, and 36-month visits, but none at the 6-year visit. HPV positivity at birth or later was unrelated to the mode of delivery. Overall, 41.4% (135/329) of the children remained negative for all oral samples collected during the follow-up.

Incident HPV infection (baseline negative) was determined for 107 (32.8%) of 326 children: 107 cases/5,754 person-months at risk (PMR), which resulted in an incidence rate of 18.6 cases/1,000 PMR. Ten children (9.4%) had multiple-type infections among this group of incident infections. Kaplan-Meier analysis showed that there were no significant differences in the acquisition of oral HPV between the different species (Appendix Figure). The incidence of new HPV genotypes during the follow-up were also investigated by HPV clades. The results indicated that none of the HPV genotypes present at birth would promote acquisition of another specific HPV genotype, not even an HPV from the same clade. However, newborns with oral HPV6 or HPV11 (n = 4) acquired only HPV16 or HPV18 genotypes. We provide the type distribution of children who were positive at 6-year visit across different time points (Appendix Table 2). The results showed that 63% (26/41) had the same genotype detectable already at birth, and 14.6% (6/41) of the children had the same genotype at some visit during the follow-up, but not at birth. Of the children, 22% (9/41) had the genotype present only at most recent (6-year) follow-up visit. Four of the children positive for HPV6 at birth still had this genotype at their 6-year follow-up visit.

**Table 2:** Duration of genotype and species-specific persistence of oral HPV infection in children*

HPV genotypes/clades	No.	Mean persistence, mo (range)
HPV6	2	19.7 (1.8–37.5)
HPV16	36	19.8 (0.1–82)
HPV18	3	11.8 (5.0–18.6)
HPV31	1	92.2 (92.2)
HPV33	3	14.2 (1.0–40.6)
HPV39	1	89.0 (89.0)
HPV58	1	88.7 (88.7)
Multiple-type infections (**>**2)	20	14.2 (1.0–91.0)
Clade A7: HPV18, 39, 45, 59, 68, 70, 85	4	31.2 (5.0–89.0)
Clade A9: HPV16, 31, 33, 35, 52, 58	41	22.8 (0.1–92.2)
Clade A10: HPV6, 11, 13, 44, 55, 74	2	19.7 (1.8–37.7)

*Persistence is defined as having >2 consecutive visit HPV-positive results for the same HPV genotype or clade. HPV, human papillomavirus.

A total of 99 children cleared their oral HPV infection during the follow-up, resulting in a clearance rate of 19.1 cases/1,000 PMR (99/5,183). The mean clearance times for clades 10 (HPV6/11 and their closest relative), 9 (HPV16 group), and 7 (HPV18) genotypes were not significantly different: 28.6, 34.2, and 30 months, respectively (Appendix Figure).

A total of 14.9% (48/323) of the children had persistent oral HPV infection. The mean time of persistence was 20.6 months (range 0.1–92.2 months). We provide type-specific HPV persistence times (Table 2). The most prevalent type to persist was HPV16, which had a persistence time of 19.8 months, followed by multiple-type infections (persistence time 14.2 months), HPV18 (persistence time 11.8 months), HPV33 (persistence time 14.2 months), and HPV6 (persistence time 19.7 months). The 6 children who had multiple-type HPV infections at birth still had them at the most recent visit. Our results show that clade α9 resulted most frequently in the full-time persistence of oral HPV infection in early childhood, followed by clade α7.

We summarized the predictors of incident, cleared, and persistent oral high-risk HPV infections in these children (Table 3). All established or implicated risk factors in our previous studies were tested as covariates, but we report only those that showed any significant predictive value (Table 3). Demographic data obtained at the 36-month visit for children did not show any association for oral HPV (Appendix Table 1). High-risk HPV seropositivity was associated with oral high-risk HPV incidence for fathers and clearance for children. Incidence rates were 3.32 (95% CI 1.24–8.91) for fathers and 5.84 (95% CI 2.09–16.32) for children. Conversely, baseline oral carriage for mothers, as well as high-risk HPV seroconversion, were associated with persistent oral high-risk HPV infection for children. Odds ratios were 1.92 (95% CI 1.35–2.74) for baseline oral carriage and 1.60 (95% CI 1.02–2.50) for high-risk HPV seroconversion.

**Table 3:** Predictors of incident, cleared, and persistent high-risk HPV infection in oral mucosa of children*

Predictor	Oral high-risk HPV infection		
	Incident, IRR (95% CI)†	Clearance, IRR (95% CI)†	Persistence,‡ OR (95% CI)†
Oral HPV DNA			
Mother	1.05 (0.49–2.27)	1.72 (0.95–3.10)	**1.92 (1.35–2.74)**
Father	1.80 (0.79–4.08)	1.60 (0.70–3.65)	1.39 (0.57–3.39)
Seropositive to high-risk HPV at baseline			
Mother	1.67 (0.67–4.12)	1.76 (0.74–4.20)	1.15 (0.75–1.76)
Father	**3.32 (1.24–8.91)**	**5.84 (2.09–16.32)**	1.25 (0.61–2.53)
Seroconversion to high-risk HPV			
Mother	2.20 (0.90–5.36)	**2.86 (1.15–7.10)**	**1.60 (1.02–2.50)**
Father	1.10 (0.57–2.10)	1.44 (0.46–4.49)	0.92 (0.39–2.17)
Child	0.83 (0.56–1.23)	1.48 (0.55–3.95)	0.93 (0.58–1.48)

*Values in bold are significant. Analyses were performed by using generalized estimating equation modeling (persistence) and Panel Poisson (incident and cleared) restricted to high-risk HPV types from species 7 (HPV genotypes: 18, 39, 45, 59, 68, 70, 85) and 9 (HPV genotypes: 16, 31, 33, 35, 52, 58). HPV70 was also included in the analyses even if labeled as a low-risk HPV genotype in the insert of the Multiplex Kit (Progen Biotechnik GmbH, https://www.progen.com). HPV70 can be a possible carcinogen (limited evidence according to International Agency for Research on Cancer classification. HPV, human papillomavirus; IRR, incidence rate ratio; OR, odds ratio. †Adjusted for age and all significant (and borderline significant) univariates in the model. ‡Binary outcome (persistent/not persistent), as defined by persistence of the 2 original HPV species (same genotype) during the follow-up.

## Discussion

HPV infections in the oral cavity have been detected in young children, but the outcome of these infections has remained unknown. We found that the prevalence of HPV and multiple-type infections was highest and the spectrum of HPV genotypes was widest at birth. The mode of delivery had no association with oral HPV carriage, and some sex differences were found in oral HPV prevalence during the early months, but not at the end of the follow-up period. Results indicate that none of the HPV genotypes present at birth would promote acquiring another specific HPV genotype, not even an HPV from the same clade. Although most of the oral HPV infections were cleared during the 6-year follow-up period, persistent oral HPV infection was found in 14.9% of these children. The 6 children who had multiple-type HPV infections at birth still harbored those infections at the most recent visit. Thus, clade α9 resulted most frequently in the full-time persistence of oral HPV infection during the early childhood, followed by α7 as the second most frequent clade.

HPV acquisition at birth has been regarded to be caused by vertical transmission, although controversial opinions have been reported (*3*,*5*,*6*). The debate is ongoing, particularly regarding the magnitude of risk, as well as route and timing, and whether mother-to-child transmission of HPV is a major infection route. Neonatal HPV infection through vertical transmission is believed to be transient, although there have been only a few follow-up studies (*5*,*11*,*12*,*22*). One of those studies showed that 37% (39/106) of nasopharyngeal aspirates of newborns were HPV positive, and concordance between HPV types in the mother (genital tract) and newborn was 69% (*12*). In a few days, HPV positivity disappeared in 38% of these newborns. However, 10.4% of the infants had the same HPV type detectable at birth and after 3 months to 3 years (*12*). Another study reported that 32% (31/98) of the children (age range 3.6 months–11.6 years) born to mothers who had cervical HPV infections at the time of delivery had HPV detectable in their oral mucosa (*11*). A total of 52% of these children had an HPV type identical with that of their mothers; HPV16/18 was most prevalent (81%).

Our results are consistent with previous results because they show that oral HPV is detectable in 22.8% of newborns. This HPV detection rate is almost identical to what Castellsague et al. reported in 2009, showing that the overall oral HPV prevalence at any visit was 18.2% during the mean follow-up of 14 months, by using PCR (MY09/MY11 primers), followed by subsequent hybridization with specific probes for HPV6, 11, 16, 18, 31, 33, and 39 (*22*). That study reported HPV positivity of 12.7% (14/110) at 6 weeks of age; in our study, HPV positivity at month 2 was 16.2% (48/296). Similar to our present results, HPV16 was the most frequent genotype, followed by HPV6/11, HPV18, and HPV31 (*22*) and in this study by HPV18, 6, 31, and 33.

Our results are derived from a longitudinal study rather than a cross-sectional study, in which autocorrelation (intrasubject variability) has been controlled by models for panel data (GEE and panel Poisson). Trottier et al. published their first results from the HERITAGE study on perinatal transmission and risk for HPV persistence in children (*23*). The design of their study is nearly identical with that of our study, but they extended sampling to conjunctival, pharyngeal, and genital sites. Their preliminary results on 75 HPV-positive participating mothers and their 67 infants sampled at birth and at 3-month visits showed that overall HPV positivity in children was 11% (range 5%–22%). Site-specific HPV positivity for conjunctival and genital areas were 4.8% and 4.8%, respectively (*23*). However, the HPV detection rate was only 8.1% for oral sites and 1.6% for oropharyngeal sites, which was lower than that reported in our study. Oral sampling (brushing of the entire oral mucosa vs. Dacron swab of buccal mucosa) and HPV amplification (nested vs. single PCR) might explain the differences in oral HPV detection rates between these 2 studies.

HPV data for mothers were not included in our report because these data have been reported in other studies (*14*,*20*,*24*,*25*). In brief, HPV DNA was detected in 17.9% of baseline oral samples from newborns and in 16.4% of maternal cervical samples (*14*). The HPV genotype-specific concordance between the newborns at delivery and the mothers was almost perfect (weighted κ = 0.988; 95% CI 0.951–0.997), but this concordance disappeared in 2 months (*14*). We have also shown that oral HPV carriage in newborns was most significantly associated with HPV presence in the placenta or cord blood (*9*,*14*). Together with these previous baseline data, our study strongly supports the hypothesis that HPV can be transmitted vertically and cause a true infection of oral mucosa of the newborn. Some of these oral HPV infections acquired at birth can also persist for years without any major clinical lesions. We reported the detection of HPV16-specific cell-mediated immunity in a small number of sexually inexperienced children from this cohort (*25*,*26*). However, we cannot determine by detection of HPV DNA the time when HPV-evoked immune recognition occurred. It has also been shown that half of healthy adults demonstrate HPV-specific cell-mediated immunity, irrespective of their partner/sexual status (*27*,*28*).

Oral HPV persistence during the 6-year follow-up period was predicted by oral HPV infection and seroconversion to high-risk HPV of the mother during the follow-up. We have recently shown that human leukocyte antigen G has a role in predicting the likelihood of the newborn for oral HPV infection at birth (*29*). However, human leukocyte antigen G had no association with HPV genotype-specific concordance between the mother and her child at birth or influence on perinatal HPV status of the child. This finding suggests that some persistent oral HPV infections after birth are not caused by vertical transmission but are acquired horizontally from the mother. This finding also indicates that transmission from a mother to her child continues during early childhood. In addition, some of the so-called persistent HPV infections could be reinfections among the family, and the incident HPV infections for a child were predicted by HPV seropositivity of the father. However, reinfection of the child needs to be further studied.

In our study, 41% of children remained constantly HPV negative during the follow-up period (<9 consequent oral samples). We do not know yet whether these children will continue to remain HPV negative later in life. We suspect that these children might be less prone to HPV infections in general, and would be interesting to evaluate again later in life.

In conclusion, our results indicate that HPV infection can be acquired nonsexually and is already common at an early age. The oral cavity is the common site of the first HPV exposure, and the mother is the most likely source of first HPV infection in her child. These results have several major implications in HPV vaccination programs. If a subgroup of children can acquire a persistent HPV infection, the timing of prophylactic HPV vaccination is imperative. Maternal HPV antibodies, irrespective of whether they are acquired by natural HPV infection or vaccination, might protect the fetus, newborn, and young child against early HPV infection. In addition, children who have persistent HPV infections (caused by immunologic tolerance) might also benefit from vaccination, as has been the case with hepatitis B virus–infected newborns or children (*30*).

Appendix Additional information on oral human papillomavirus infection in children during the first 6 years of life.
