# Supplementary material for: Oral Human Papillomavirus Infection in Children during the First 6 Years of Life, Finland
Source: Emerg Infect Dis. 2021 Mar;27(3):759–66. doi: 10.3201/eid2703.202721 (PMC7920652; doi:10.3201/eid2703.202721)
Supplement: Appendix — Additional information on oral human papillomavirus infection in children during the first 6 years of life. [file 20-2721-Techapp-s1.pdf]

Article DOI: https://doi.org.10.3201/eid2703.202721

Oral Human Papillomavirus Infection in Children during the First 6 Years of Life

Appendix

Appendix Table 1. Demographic data for 203 children tested for human papillomavirus at 36-month visit whose parents completed a questionnaire

Background information	No. (%)
Order of the child in family, n = 328	
First born	178 (54.3)
Second child	73 (22.1)
Third child	2 (0.6)
Hand warts, n = 203	
Yes	6 (3.0)
No	197 (97)
Warts in sole, n = 203	
Yes	2(1.0)
No	201 (99)
Warts in trunk, n = 203	
Yes	1 (0.5)
No	202 (99.5)
Mollusca contagiosum, n = 203	
Yes	18 (8.9)
No	185 (91.1)
Allergy, n = 199	
Yes	146 (73.4)
No	53 (26.6)
Atopic symptoms, n = 199	
Yes	146 (73.4)
No	53 (26.6)
Any medication, n = 200	
Yes	13 (6.5)
No	187 (93.5)
Corticosteroids	
Yes	7 (3.5)
No	193 (96.5)
Other medication	
Yes	6 (3.0)
No	194 (97.3)
Diseases diagnosed, n = 200	
Asthma	
Yes	9 (4.5)
No	191 (95.5)
Other	
Yes	3 (1.5)
No	197 (98.5)
Operations, n = 200	
Yes	39 (19.5)
No	161 (80.5)

Appendix Table 2. Oral HPV types at follow-up visits of 41 children positive for HPV at the most recent (6 y) follow-up visit*

Child no.	HPV type at birth	HPV type at 3 d	HPV type at 1 mo	HPV type at 2 mo	HPV type at 6 mo	HPV type at 12 mo	HPV type at 24mo	HPV type at 36 mo	HPV type at 6 y
1	33, 59	–	33	HPV–	HPV–	–	–	–	33, 59
2	39	–	–	–	–	–	–	–	39
3	58	HPV–	HPV–	HPV–	16, 70	HPV–	HPV–	HPV–	58
4	16, 39	16	HPV–	HPV–	HPV–	–	–	–	39
5	–	HPV–	HPV–	HPV–	16, 18	16	HPV–	HPV–	16
6	31	HPV–	HPV–	HPV–	HPV–	HPV–	70	18	31
7	33, 59, 66	HPV–	HPV–	HPV–	HPV–	HPV-	HPV–	HPV–	33, 59, 66
8	HPV–	6	HPV–	HPV–	70	HPV–	...	HPV–	16
9	HPV–	HPV–	HPV–	HPV–	59, 66	HPV–	HPV–	16	16
10	31	6	33	HPV–	HPV–	HPV–	HPV–	HPV–	31
11	6	HPV–	HPV–	HPV–	HPV–	HPV–	6	HPV–	6
12	33	HPV–	HPV–	HPV–	HPV–	HPV–	HPV–	16	33
13	33, 59	HPV–	66	HPV–	HPV–	HPV–	HPV–	HPV–	33, 59
14	16, 33, 59	HPV–	16	18	HPV–	HPV–	HPV–	HPV–	33, 59
15	33, 59	–	HPV–	HPV–	16, 18	HPV–	HPV–	HPV–	33, 59
16	HPV–	HPV–	HPV–	HPV–	HPV–	18	HPV–	HPV–	33
17	HPV–	HPV–	HPV–	HPV–	HPV–	52	HPV–	HPV–	16
18	45, 66	HPV–	HPV–	HPV–	HPV–	HPV–	HPV–	HPV–	6, 45, 66
19	16	HPV–	16	–	HPV–	HPV–	33	HPV–	16
20	HPV–	HPV–	HPV–	HPV–	HPV–	6	HPV–	HPV–	16
21	16	HPV–	HPV–	HPV–	HPV–	HPV–	HPV–	HPV–	16
22	6	HPV–	HPV–	6	HPV–	–	–	HPV–	6
23	HPV–	HPV–	–	–	–	–	–	–	16
24	16	HPV–	16	16	HPV–	HPV–	HPV–	HPV–	16
25	16	HPV–	16	16	HPV–	HPV–	16	16	16
26	16	HPV–	16	70	HPV–	HPV–	–	–	16
27	18	HPV–	16	HPV–	HPV–	HPV–	16	16	18
28	11	HPV–	18	HPV–	HPV–	HPV–	HPV–	HPV–	18
29	HPV–	HPV–	HPV–	18	HPV–	HPV–	HPV–	HPV–	16
30	HPV–	–	16	HPV–	HPV–	HPV–	HPV–	HPV–	16
31	HPV–	HPV–	16	16	HPV–	16	HPV–	HPV–	16
32	16	HPV–	16	HPV–	16	HPV–	HPV–	HPV–	16
33	6	HPV–	HPV–	HPV–	HPV–	HPV–	16, 66	HPV–	6
34	HPV–	–	HPV–	HPV–	HPV–	–	–	–	31
35	HPV–	16	HPV–	6	HPV–	HPV–	HPV–	HPV–	16
36	16	HPV–	HPV–	HPV–	HPV–	16	HPV–	16	16
37	6	HPV–	–	–	–	–	–	–	6
38	16	HPV–	–	16	6	HPV–	16	HPV–	16
39	HPV–	HPV–	HPV–	HPV–	16, 59	–	–	–	16
40	45	HPV–	HPV–	HPV–	HPV–	HPV–	16	16	45
41	HPV–	HPV–	16	HPV–	16	HPV–	HPV–	HPV–	16

*HPV, human papillomavirus; –, sample not available..

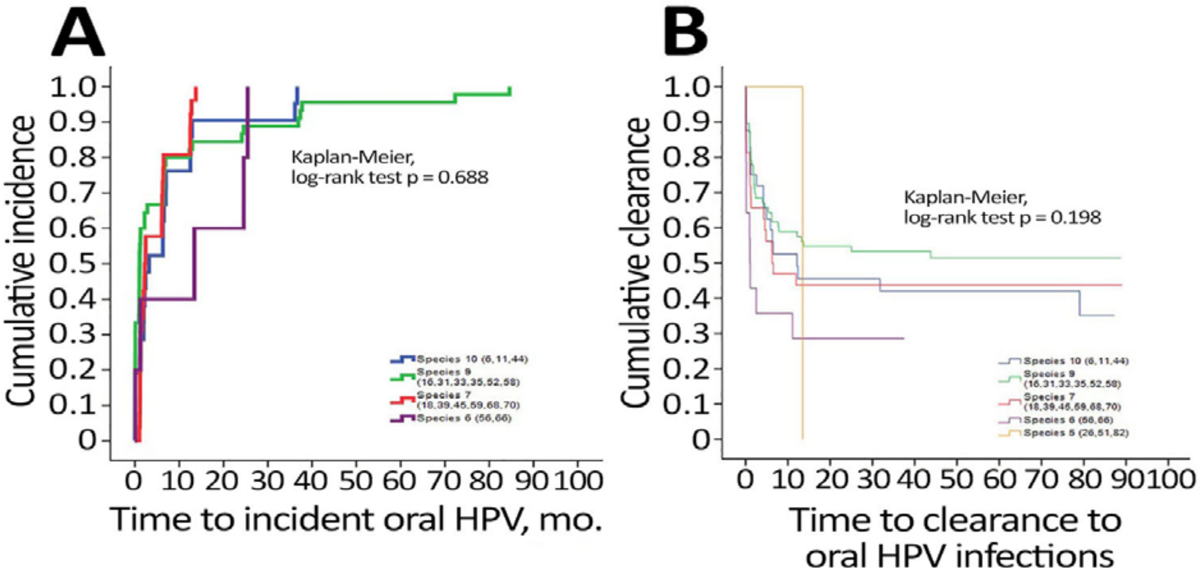

Appendix Figure. A) Cumulative incidence and B) clearance by species 5, 6, 7, 9, or 10 of oral HPV infection among children in the Finnish Family HPV study. No significant differences were seen in either acquisition or clearance of oral HPV between different species. HPV, human papillomavirus.
